# A YOLO-based AI system for classifying calcifications on spot magnification mammograms

**DOI:** 10.1186/s12938-023-01115-w

**Published:** 2023-05-27

**Authors:** Jian-Ling Chen, Lan-Hsin Cheng, Jane Wang, Tun-Wei Hsu, Chin-Yu Chen, Ling-Ming Tseng, Shu-Mei Guo

**Affiliations:** 1 Department of Radiology, Far Eastern Memorial Hospital, No. 21, Sec. 2, Nanya S. Rd., Banciao Dist., New Taipei City, 220 Taiwan; 2 Department of Radiology, Taipei Veterans General Hospital, No. 201, Sec. 2, Shipai Rd., Beitou Dist., Taipei City, 112 Taiwan; 3 Institute of Computer Science and Information Engineering, National Cheng Kung University, No. 1, University Rd., Tainan City, 701 Taiwan; 4 Department of Radiology, National Taiwan University College of Medicine, No. 1, Jenai Rd., Taipei City, 100 Taiwan; 5 Department of Nurse-Midwifery and Women Health, and School of Nursing, College of Nursing, National Taipei University of Nursing and Health Sciences, No. 365, Mingde Rd., Beitou Dist., Taipei City, 112 Taiwan; 6 Department of Biomedical Imaging and Radiological Sciences, National Yang Ming Chiao Tung University, No. 155, Sec. 2, Linong St., Beitou Dist., Taipei City, 112 Taiwan; 7 Department of Radiology, Chi-Mei Medical Center, No. 901, Zhonghua Rd. Yongkang Dist., Tainan City, 710 Taiwan; 8 Comprehensive Breast Health Center, Taipei-Veterans General Hospital, No. 201, Sec. 2, Shipai Rd., Beitou Dist., Taipei, 112 Taiwan; 9 Department of Surgery, Taipei Veterans General Hospital, No. 201, Sec. 2, Shipai Rd., Beitou Dist., Taipei, 112 Taiwan; 10 Department of Surgery, School of Medicine, National Yang Ming Chiao Tung University, No. 155, Sec. 2, Linong St., Beitou Dist., Taipei, 112 Taiwan

**Keywords:** Breast cancer, Artificial intelligence, Mammography, Calcifications, Biopsy

## Abstract

**Objectives:**

Use of an AI system based on deep learning to investigate whether the system can aid in distinguishing malignant from benign calcifications on spot magnification mammograms, thus potentially reducing unnecessary biopsies.

**Methods:**

In this retrospective study, we included public and in-house datasets with annotations for the calcifications on both craniocaudal and mediolateral oblique vies, or both craniocaudal and mediolateral views of each case of mammograms. All the lesions had pathological results for correlation. Our system comprised an algorithm based on You Only Look Once (YOLO) named adaptive multiscale decision fusion module. The algorithm was pre-trained on a public dataset, Curated Breast Imaging Subset of Digital Database for Screening Mammography (CBIS-DDSM), then re-trained and tested on the in-house dataset of spot magnification mammograms. The performance of the system was investigated by receiver operating characteristic (ROC) analysis.

**Results:**

We included 1872 images from 753 calcification cases (414 benign and 339 malignant) from CBIS-DDSM. From the in-house dataset, 636 cases (432 benign and 204 malignant) with 1269 spot magnification mammograms were included, with all lesions being recommended for biopsy by radiologists. The area under the ROC curve for our system on the in-house testing dataset was 0.888 (95% CI 0.868–0.908), with a sensitivity of 88.4% (95% CI 86.9–8.99%), specificity of 80.8% (95% CI 77.6–84%), and an accuracy of 84.6% (95% CI 81.8–87.4%) at the optimal cutoff value. Using the system with two views of spot magnification mammograms, 80.8% benign biopsies could be avoided.

**Conclusion:**

The AI system showed good accuracy for classification of calcifications on spot magnification mammograms which were all categorized as suspicious by radiologists, thereby potentially reducing unnecessary biopsies.

## Background

Breast cancer is the most common malignancy and one of the leading causes of cancer death in women worldwide [1]. For decades, mammography has been widely used as both a diagnostic tool and a screening test. Mammography is reliable in detection of microcalcifications which may be one of the major imaging signs of breast cancers. When screening mammography shows indeterminate appearing microcalcifications, diagnostic spot magnification mammograms will be performed thereafter, since the spot magnification mammograms show better spatial resolution and higher signal-to-noise ratio to evaluate the morphology, distribution and extension of the indeterminate calcifications than screening mammography does, with management based on the most suspicious features. However, the decision of categorization is still dependent on subjective interpretation by radiologists. Although malignant rates vary [2], calcifications of categories 4 (suspicious) and 5 (highly suggestive of malignancy) as per the American College of Radiology Breast Imaging Reporting and Data System (BI-RADS) are recommended for biopsy. The positive predictive value (PPV) based on recommendation for tissue diagnosis is approximately 20–40% [3–5]. That is, a large proportion of breast biopsies yield benign results, and the high false-positive rate increases patient anxiety and the workload of health-care systems.

Deep learning-based artificial intelligence (AI) systems have been applied in breast imaging. Many studies have shown that AI systems may reduce workload in screening mammograms [6–13]. However, few related studies have focused on spot magnification mammograms, which are often performed for indeterminate calcifications on screening mammograms. Stelzer et al. investigated texture analysis combined with a machine learning to predict malignancy in suspicious mammographic calcifications on one craniocaudal magnification view per patient, with their approach avoiding unnecessary biopsies by 37.1–45.7% [14].

AI with deep learning for mammographic images require considerable computation power and time due to the high image resolution. In particular, intense noise decreases a learning machine’s performance. You Only Look Once (YOLO) is an implementation of convolutional neural network (CNN) and is a state-of-the-art, real-time object detection system introduced in 2016 [15]. This model provides faster and more accurate objection detection and classification than other CNN models [16]. Several studies have reported the good performance of the YOLO-based system for breast lesion detection or simultaneous detection and classification on full-field mammograms [17–21]. Baccouche et al. indicated that the YOLO-based model demonstrated better performance for the detection and classification of mass lesions than for the detection and classification of calcifications on mammograms because calcifications show higher variety of shapes, distributions and not as solid as compared with masses [22].

In this study, we further developed a new AI system using a YOLO-based algorithm, named YOLO-adaptive multiscale decision fusion (YOLO-AMDF), with a deep ensemble module, to classify calcifications on spot magnification mammograms [23]. Our private dataset comprised cases which were all evaluated as “suspicious” by radiologists and sent for biopsy. This made the classification task more challenging than usual screening population. The model’s lesion classification performance was evaluated on both a public dataset of full-field digitized mammograms and an in-house dataset of spot magnification mammograms with biopsy-confirmed results of calcifications all categorized as “suspicious” by radiologists. The model showed better performance than the original YOLO with good accuracy for classification of calcifications on spot magnification mammograms, thereby potentially reducing unnecessary biopsies.

## Results

### Basic characteristics

The characteristics of the selected cohort are summarized in Tables 1 and 2. The selected dataset from the public dataset Curated Breast Imaging Subset of Digital Database for Screening Mammography (CBIS-DDSM) [24] contained 753 cases with calcifications (414 benign and 339 malignant) with 1872 mammogram images. We split them into 80% (602 cases) for training and 20% (151 cases) for testing the proposed algorithm in this study.

**Table 1:** Number of images in the train and test sets of CBIS-DDSM and in-house dataset

Dataset	Splits	Cases (*n*)		Images (*n*)	
		Benign	Malignant	Benign	Malignant
CBIS-DDSM	Train	329	273	1002	544
	Test	85	66	197	129
	Total	414	339	1199	673
In-house	Train	382	154	761	308
	Test	50	50	100	100
	Total	432	204	861	408

CBIS-DDSM, Curated Breast Imaging Subset of the Digital Database for Screening Mammography

**Table 2:** Summary of characteristics of the in-house dataset

Characteristics	No. of cases
No. of spot magnification images*	1269
Age at examination (y)^†^	29–81 (54.4 ± 8.42)
Mean time interval between image acquisition and biopsy (d)^‡^	48.71 (46.3, 51.1)
BI-RADS assessment	*n* (%)
4A (low suspicion for malignancy)	435 (68.4)
4B (moderate suspicion for malignancy)	178 (27.99)
4C (high suspicion for malignancy)	23 (3.61)
Histologic type of malignancy	*n* (%)
Invasive ductal carcinoma	61 (29.9)
Invasive lobular carcinoma	3 (1.5)
Ductal carcinoma in situ	140 (68.6)

Except where indicated, data are numbers of cases, with percentages in parenthesis

BI-RADS, Breast Imaging Reporting and Data System

*Three of the cases had only one image for each

^†^Data in parenthesis are means ± standard deviations

^‡^Data in parenthesis are 95% confidence intervals

Our in-house dataset contained 636 cases (432 [67.92%] benign and 204 [32.08%] malignant) with 1269 images. All patients were women aged 29–81 years who had suspicious appearing calcifications on diagnostic spot magnification mammograms with biopsy pathology reports available. Three patients with only one view of magnification mammograms were also included because the biopsied targets could be confidently confirmed to be the same calcifications reported on that single magnification view. The mean time interval between the diagnostic mammography and biopsy was 48.71 days. Most cases were interpreted as BI-RADS category 4A (*n* = 435, 68.4%). Because of the relatively small number of cases in our in-house dataset, we randomly split cases into 85% (536 cases) for the training set and 15% (100 cases) for the test set to enrich the proportion of training material.

### Performance of the YOLO model and modified YOLO-based AI system

The results of performance of AI are summarized in Table 3. To explore how the proposed modification of YOLO-AMDF affected the performance of the YOLO-baseline, we compared the performance of these two models on our in-house dataset with single view of mammogram, which included craniocaudal, mediolateral oblique, and mediolateral views. On our in-house dataset, the AUC of YOLO-AMDF was 0.83 (95% CI 0.81–0.85) and accuracy was 79.4% (95% CI 77.1–81.7%); the AUC of YOLO-baseline was 0.766 (95% CI 0.732–0.8) and accuracy was 74.5% (95% CI 71.2–77.8%). Using the YOLO-AMDF module, the improvement in the mean AUC was 8.3% (*P* < 0.05).

**Table 3:** Diagnostic accuracy of for differentiating benign and malignant calcifications on spot magnification mammogram of in-house dataset

	AUC	Sen %	Spec %	Acc %	PPV %
YOLOv4-baseline on single view	0.766 (0.732–0.800)	78.6 (74.9–82.3)	70.4 (63.4–77.4)	74.5 (71.2–77.8)	77.1 (73.7–80.5)
YOLO-AMDF on single view	0.830 (0.810–0.850)	78.7 (76.1–81.3)	80.2 (77.9–82.5)	79.4 (77.1–81.7)	80.0 (77.8–82.2)
Proposed AI system on single view	0.867 (0.851–0.883)	83.0 (80.2–85.8)	81.0 (77.9–84.1)	82.0 (79.8–84.2)	82.3 (79.9–84.7)
Proposed AI system on two views	0.888 (0.868–0.908)	88.4 (86.9–89.9)	80.8 (77.6–84.0)	84.6 (81.8–87.4)	83.3 (80.8–85.8)

The numbers in parentheses indicate 95% confidence intervals

AUC, areas under the receiver operating characteristics curve; Sen, sensitivity; Spec, specificity; Acc, accuracy; PPV, positive predictive value; YOLOv4, You Only Look Once version 4; YOLO-AMDF, YOLO-Adaptive Multiscale Decision Fusion

To confirm the effectiveness of our system, we also validated it on CBIS-DDSM and compared it with other work on this dataset. The results are summarized in Table 4. Our system achieves the AUC of 0.847 and the accuracy rate of 79.5%, outperforming the result of others.

**Table 4:** Performance of proposed system and other algorithms on CBIS-DDSM dataset

Methods (single view)	AUC	Sen %	Spec %	Acc %	PPV %
Proposed AI system	0.847	68.2	86.8	79.5	77.2
YOLOv4-baseline	0.76	66.8	75.1	71.8	63.7
AlexNet	0.711	53.5	75.1	66.6	58.5
VGG-16	0.713	58.9	75.6	69.0	61.3
CSPDarknet53	0.660	41.1	82.7	66.3	60.9
CSPDarknet53 + AMDF	0.759	51.2	85.8	72.1	70.2

AUC, areas under the receiver operating characteristics curve; Sen, sensitivity; Spec, specificity; Acc, accuracy; PPV, positive predictive value; YOLOv4, You Only Look Once version 4

**Fig. 1:**
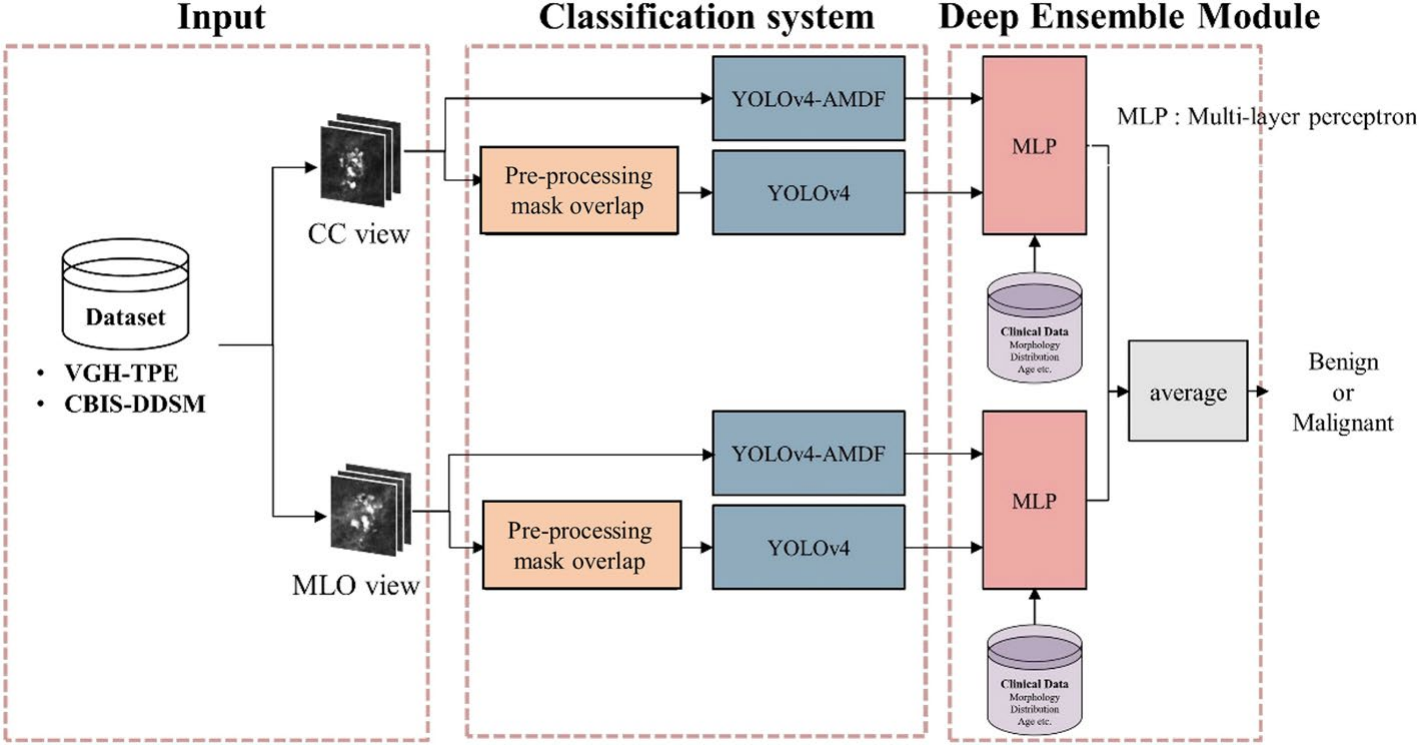
The proposed classification system of calcifications on mammograms. It consists of three stages: input, detecting calcification features using YOLOv4, and classifying using multilayer perceptron in deep ensemble module. CBIS-DDSM, Curate Breast Imaging Subset of Digital Database for Screening Mammography; CC, craniocaudal; MLO, mediolateral oblique; YOLOv4, You Only Look Once version 4; AMDF, adaptive multiscale decision fusion

When evaluating the performance of the overall proposed AI system incorporating the YOLO-AMDF and deep ensemble module in the model (Fig. 1) on the in-house dataset with two views, that is, the average prediction score of both views (both craniocaudal and mediolateral oblique views, or both craniocaudal and mediolateral views) for each case, the overall AUC in the five holdout validations was 0.888 (95% CI 0.868–0.908), and the accuracy was 84.6% (95% CI 81.8–87.5%). The sensitivity was 88.4% (95% CI 86.9–89.9%), and the specificity was 80.8% (95% CI 77.6–84.0%). Therefore, in our test set, 80.8% benign biopsies could be avoided.

## Discussion

In this study, we developed a proposed AI algorithm for classifying calcifications on spot magnification mammograms assessed as BI-RADS category 4 by radiologists, which yielded an AUC of 0.888 for our in-house dataset. To our knowledge, this study is the first attempt to use the YOLO-based model to classify suspicious appearing calcifications on spot magnification mammograms.

The previously reported studies have explored the application of AI systems on the workflow of breast cancer screening [6–13, 25–29]. Most of the algorithms were developed on full-field digital mammograms and cannot be directly applied on magnifications mammograms. However, in a standard diagnostic workflow for calcifications on full-field mammograms, spot magnification views for indeterminate calcifications are usually the last decisive images undertaken before biopsy. Therefore, our algorithm was developed to assist in classification of calcifications on spot magnification views. And to provide a training dataset with better quality, we used ground truth labels with bounding box annotation for the calcifications on images.

The success of deep CNN in the 2012 ImageNet Large Scale Visual Recognition Challenge [30] triggered an interest in the development of better automated image analysis methods. CNN-based methods not only greatly improve classification performance but also eliminate the need to manually select distinguishable features. In this study, we chose YOLO as our baseline deep learning CNN model because it has better detection than other object detection CNN techniques, such as Fast R-CNN and Retina-Net. YOLO is open source, efficient and suitable for single GPU training [17]. As a computer-aided diagnosis system, the basic aim is fast diagnosing suspicious regions captured from CC and MLO views by radiologists. Our system comprises YOLOv4 and YOLOv4-AMDF with 59.570 and 59.573 billion floating-points operations per second (BFLOPs), respectively. MLP ensemble module is less than 0.001 BFLOPs. Hence, the computational complexity of our system is about 119.143 BFLOPs. Our computer-aided diagnosis system takes 0.430 s to diagnose one case (with both CC and MLO views) using a NVIDIA GeForce RTX 3060 GPU, which can provide radiologists with AI-diagnosed results immediately.

In our study, the proposed AI system with modification of the AMDF model combining deep ensemble module enhanced the performance of YOLO for the classification of calcifications, in both the public dataset CBIS-DDSM and our in-house datasets. However, whether the performance of the model is comparable to the regular non-magnified digital mammograms needs further validation.

Among the AI systems developed for mammograms, some were trained on a single view and some on multiple views. To be in line with our clinical practice, in our study the system was trained and validated on single view, while average scores of both CC and MLO views for each case were used at exploring system performance on combining these two views. The results showed that slightly higher AUC (0.888, 95% CI 0.868–0.90) was achieved when using two views than when using only one view (0.867, 95% CI 0.851–0.883) though not reaching statistical significance. Khan et al. [31] proposed a feature fusion strategy of four views to build a classification model and concluded that multi-view feature fusion-based system is more efficient than single view-based system. Yang et al. [32] proposed enhanced multi-view DNN architecture MommiNet to perform joint ipsilateral and bilateral analysis on mammograms and showed great potential for mass malignancy classification. In most cases of spot magnification mammography, bilateral views of the breasts are not available. However, future work on processing multiple views of mammograms of each case during the development of algorithm may be worth further investigation.

This in-house dataset comprised BI-RADS category four cases, which means calcifications in all these cases were evaluated as “suspicious” by radiologists and sent for biopsy. Thus, the discrimination of breast cancer with calcifications in this population was more challenging than for the overall screening population, which generally yields negative or benign results. Our algorithm demonstrated good performance in discriminating these category 4 calcifications. Its application in the clinical setting may assist in discrimination of BI-RADS 4 calcifications during diagnostic workup and thus reduce unnecessary biopsies.

Although interobserver and intra-observer variability in mammographic interpretation is substantial [33, 34] and classification of breast calcifications based on the BI-RADS descriptors of morphology and distribution had been shown to have varied likelihood of malignancy [2, 35], descriptors for suspicious calcifications remain a core component of BI-RADS lexicon. We ensembled these imaging descriptors through MLP, and the whole proposed AI system achieved better performance by correctly classifying some cases that were difficult to distinguish by using YOLO-baseline and YOLO-AMDF. We recorded the descriptors from the original reports of radiologists to reflect the real-world practice as much as possible. Given the existence of interobserver and intra-observer variability in descriptors, the weights of this MLP might change correspondingly to the experience of radiologists. Further studies should investigate more valuable clinical characteristics as ensemble models or multi-tasking learning module utilizing richer information such as prior mammograms, age, family history, symptoms, and BI-RADS assessment.

Yoon et al. [12] found that calcifications with same morphology or BI-RADS assessment featured significantly higher positive predictive value (PPV) when they had positive AI-CAD scores than those with negative AI-CAD scores. Our results also revealed that even though the algorithm only processed the most recent spot magnification mammograms, while radiologists could obtain other information such as patient history or series of previous mammograms if available, the proposed AI system still differentiated benign and malignant calcifications for the BI-RADS 4 calcifications interpreted by radiologists. The performance of this system indicates that AI systems may assist radiologists in decision-making and thereby potentially reduce unnecessary biopsies. However, further investigation is needed to determine alternative imaging surveillance strategies for possible false-negative prediction when assessment of AI and humans is discordant.

This study has some limitations. First, this was a retrospective study from a selective population, since we only included cases of BI-RADS 4 in our in-house dataset with a small sample size enriched with cancer cases. Second, the patient cohort comprised a purely Asian women population, which may limit direct application to a more racially diverse population. Third, the original assessment of BI-RADS category 4 was reported from radiologists of different levels of experience in breast imaging, however, this reflects our routine clinical practice. Fourth, we did not perform a double reading study for the same test sets to compare the performance between AI stand-alone and double reading by radiologists, and did not validate the performance on an independent patient cohort. Therefore, the reported performance is related to a specific study setting.

In the future, extended studies may be needed to investigate the performance of this algorithm on regular full-field digital mammograms or digital breast tomosynthesis, explore valuable clinical characteristics for designing ensemble models, and examine the implementation of the algorithm in clinical workflow.

## Conclusion

Our proposed AI algorithm based on You Only Look Once model that was trained on public and private datasets with ground truth for differentiating benign and malignant calcifications on spot magnification mammograms, and the AI system showed good accuracy for classification of calcifications on spot magnification mammograms which were all categorized as suspicious by radiologists, thereby potentially reduce unnecessary biopsies. Prospective studies are needed to investigate how the potential benefits of AI translate into clinical practice.

## Methods

This retrospective study was approved by the institutional review board of our hospital and the requirement for informed consent was waived.

### Data collection

#### Public dataset

The public dataset Curated Breast Imaging Subset of Digital Database for Screening Mammography (CBIS-DDSM) is the updated version of the DDSM which contains a subset of the original DDSM images in Digital Imaging and Communications in Medicine (DICOM) format and pathologic diagnoses [24]. We included only calcification cases from the dataset and split them into 80% for training and 20% for testing the proposed algorithm in this study.

#### In-house dataset

For the in-house dataset, the data of eligible patients between January 2016 and September 2020 were retrieved from a dedicated patient registry of patients undergoing stereotactic vacuum-assisted breast biopsy or mammography-guided needle localization for subsequent excisional biopsy of suspicious calcifications on spot magnification mammography in our hospital. Demographic, radiological, and histopathological data were retrieved from our hospital’s records. The interpretation of spot magnification by radiologists was based on Breast Imaging Reporting and Data System (BI-RADS) by American College of Radiology (ACR) [3] and the suspicious appearing calcifications were assessed as BI-RADS category 4 (suspicious) and subdivided into BI-RADS 4A, low suspicious for malignancy (> 2% to ≦ 10%), BI-RADS 4B, moderate suspicion for malignancy (> 10% to ≦ 50%), and BI-RADS 4C, high suspicion for malignancy (> 50% to < 95%). Malignant pathologies included ductal carcinoma in situ, microinvasive carcinoma, invasive ductal carcinoma and invasive lobular carcinoma; all other pathologies were considered benign. Imaging data of the included patients comprised both craniocaudal and mediolateral oblique or mediolateral spot magnification views. Three patients with only one view of magnification mammograms were also included because the biopsied targets could be confidently confirmed to be the same calcifications reported on that single magnification view. Two radiologists with 10 and 20 years of experience in breast imaging retrospectively annotated the region of interest manually and surrounding the area containing biopsied calcifications after reviewing the diagnostic spot magnification and biopsy images. Because of the relatively small number of cases in our in-house dataset, we randomly split cases into 85% for the training set and 15% for the test set to enrich the proportion of training material.

### Development of the AI system

The framework of our proposed AI system and YOLO (referred to as YOLO-baseline) system is illustrated in Fig. 1. It consists of three stages: input the public and in-house dataset to the system, to detect and classify the calcification features using YOLO-baseline and the modification of YOLO as deep learning model in the classification algorithm; to make final classification of the calcifications using multilayer perceptron (MLP) in the deep ensemble module.

**Fig. 2:**
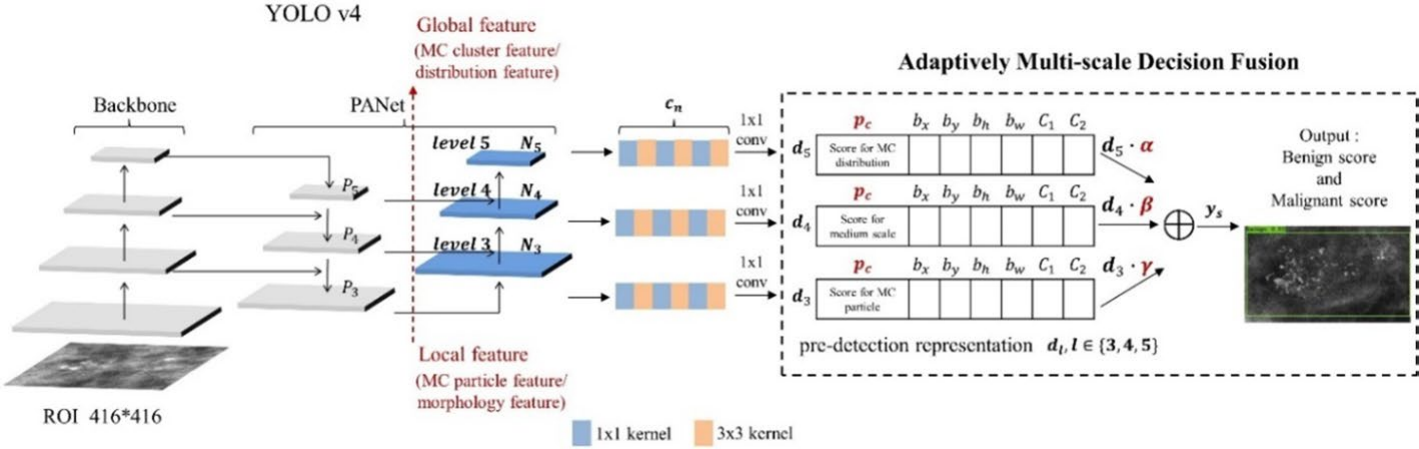
Examples of pre-processed mask overlap images. CC, craniocaudal view; MLO, mediolateral oblique view

The overall architecture is demonstrated in Fig. 2, with *N*3, *N*4, and *N*5 denoting the newly generated-feature maps corresponding to levels 3–5 of PANet [36], which is the neck architecture for feature aggregation in the original YOLO framework. The original mammograms without preprocessing were used as input of YOLO-AMDF. Finally, we classified the calcifications using multilayer perceptron (MLP) in the deep ensemble module. The MLP input comprised two parts: the confidence scores of output from the deep learning models, and the radiologists’ descriptors about morphology and distribution of target calcifications. The details of the proposed framework including preprocessing mask overlap, YOLO-AMDF and deep ensemble module are described in following sections.

### Preprocessing mammograms

**Fig. 3:**
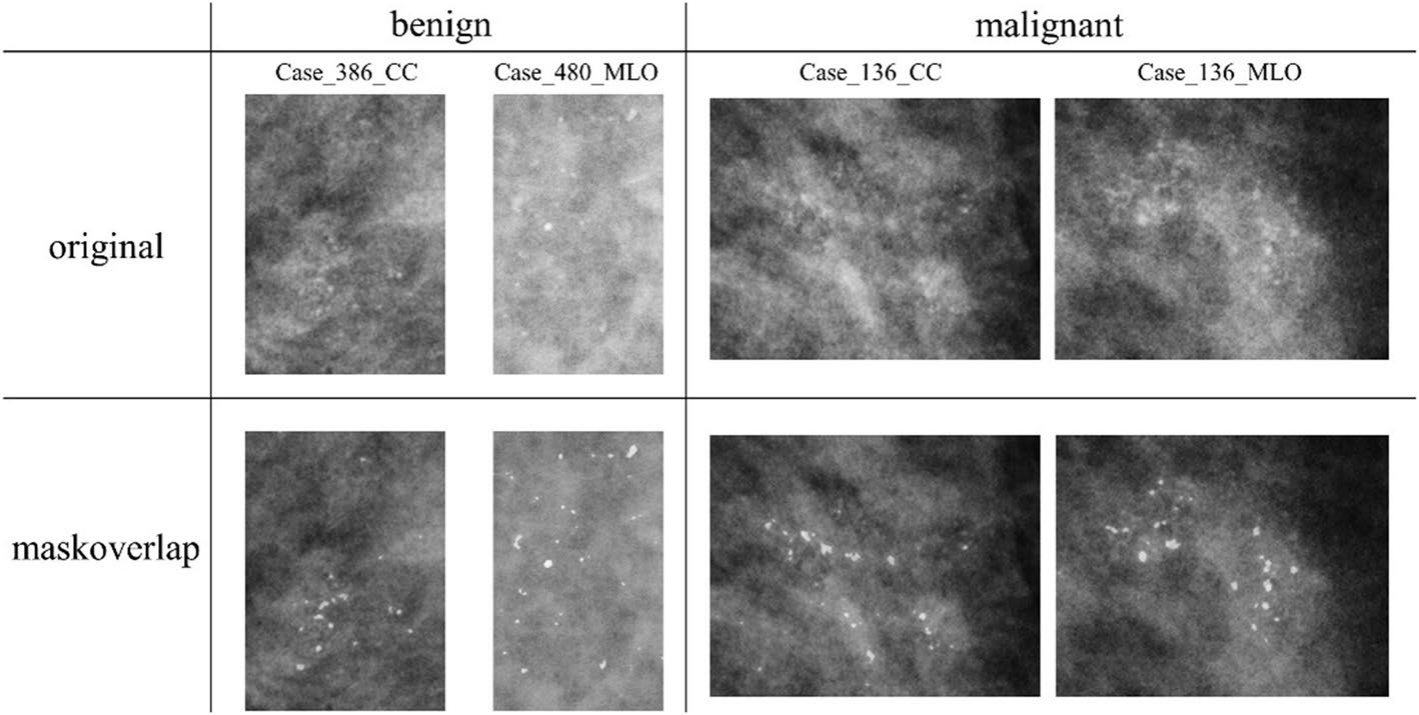
YOLOv4-AMDF architecture. The backbone and feature aggregation parts of YOLOv4 are combined with the proposed AMDF module for fusion predetection representation dl of each scales, where *p*_*c*_ denotes the objectness prediction of the corresponding levels, *b*_*x*_, *b*_*y*_, *b*_*h*_, *b*_*w*_ are the bounding box offsets, *c*_1_, *c*_2_ represent the class predictions, $\alpha$, $\beta$, $\gamma$ are reliabilities of each scale and ⊕ denotes pixel-wise addition. *ys* is the tensor for final prediction. YOLO v4, You Only Look Once version 4; MC, microcalcification; ROI, region of interest

In the stage of the classification system, we used the preprocessing mask overlap mammograms to increase the contrast between the calcifications and the background breast tissue (examples shown in Fig. 3) and used as YOLO-baseline input [37].

The procedures of preprocessing mask overlap include: first, the original image was sent through a 3 × 3 median filter to reduce noise. Next, gamma correction was performed to increase the contrast between the calcifications and remaining breast tissue. The top-hat algorithm was used to extract the calcifications and return the binary segmentation mask of calcifications. Finally, the mask and the original image were superimposed to obtain the preprocessing mammograms.

### Image data augmentation

In this research, we used three data augmentation methods to increase the training set:

1. *Image color data augmentation*. We change image saturation and exposure in [1.0/saturation, saturation] times and [1.0/exposure, exposure] times.
2. *Image transformation data augmentation*. We randomly resize the image between 0.7× and 1.3× in width and height. Also, we randomize the input size every ten iterations. We set the random coefficient as 1.4 so the input size will be set between 1.4× or $\frac{1}{1.4}$× every ten iterations.
3. *Mosaic data augmentation*. Mosaic data augmentation combines 4 training images into one. It is the new data augmentation technique introduced in YOLOv4.”

### Architecture modification of YOLO

Our system used a proposed adaptive multiscale decision fusion (AMDF) module with a pyramid architecture to correlate the features of calcifications between morphology and distribution based on YOLO (“YOLO-AMDF”) [22]. The original YOLO framework includes three parts: the backbone, neck, and head. CSPDarknet-53 is the main characteristic of the YOLO backbone. The CBM, which is a combination of convolution layer (C), batch normalization (B), and Mish activation function (M), was the input of CSPDarknet53. CSPDarknet53 divides the feature maps into two parts. In the first part, the gradient changes from the beginning to the end are recorded into the feature map, which reduces the number of calculations, and memory cost and ensures high accuracy. The second part includes the ResNet skip connections. Finally, the first part is concatenated with the feature map generated in the second part. In the neck, the PANet is used in YOLO. PANet employs bottom path augmentation with prior local convolution layers through the upsampling operation to shorten the information path and enhance the feature pyramid with accurate localization signals existing at low levels. In the head of YOLO, the feature layer is detected and regressed through the convolution layer and anchor boxes are generated with class probabilities and bounding box offsets. The overall architecture is demonstrated in Fig. 3 of the formal main text, with *N*_3_, *N*_4_, and *N*_5_ denoting the newly generated-feature maps corresponding to levels 3–5 of PANet. The network predicts three bounding boxes at each scale. These predictions are encoded as an *Nl* × *Nl* × [3 × (1 + 4 + 2)] tensor, where *l* ∈ 3, 4, 5. We obtain the predictions of these three feature maps after YOLO’s convolutional layers *cn* and define the tensor as predetection representation *d*, where *l* ∈ 3, 4, 5. Our decision fusion strategy can be represented as follows:

$$ys = \alpha d3 + \beta d4 + \gamma d5,$$

where *ys* is the weighted sum of class probabilities for diagnosis results from small to large scopes with *α*, *β*, and *γ* being their corresponding reliabilities. We adopted the softmax-based weighted fusion approach proposed by Liu et al. [38] and Wang et al. [39]. The general forms of the coefficients *α*, *β* and *γ*, can be formulated by introducing three real parameters *λα*, *λβ*, and *λγ* as follows:

$$\alpha =\frac{{e}^{{\lambda }_{\alpha }}}{{e}^{{\lambda }_{\alpha }}+{e}^{{\lambda }_{\beta }}+{e}^{{\lambda }_{\gamma }}}, \alpha +\beta +\gamma =1 \quad \mathrm{and}\quad \alpha ,\beta ,\gamma \in \left[{0,1}\right].$$

Similarly, two more equations are used for *β* and *γ*. The real parameters *λα*, *λβ*, and *λγ* can be well learned through back-propagation. We use *ys* as the tensor for final prediction. Following the back-propagation process, the gradient of feature map *Nl* can be obtained by the chain rule:

$$\frac{\partial L}{\partial {N}_{l}}=\frac{\partial L}{\partial {y}_{s}}\cdot \frac{\partial {y}_{s}}{\partial {d}_{l}}\cdot \frac{\partial {d}_{l}}{\partial {c}_{n}}\cdot \frac{\partial {c}_{n}}{\partial {N}_{l}}.$$

Note that $\frac{\partial L}{\partial {y}_{s}}$ in this equation shares the same value among all levels. Furthermore, $\frac{\partial {y}_{s}}{\partial {d}_{l}}$ equals to the corresponding reliabilities.

**Fig. 4:**
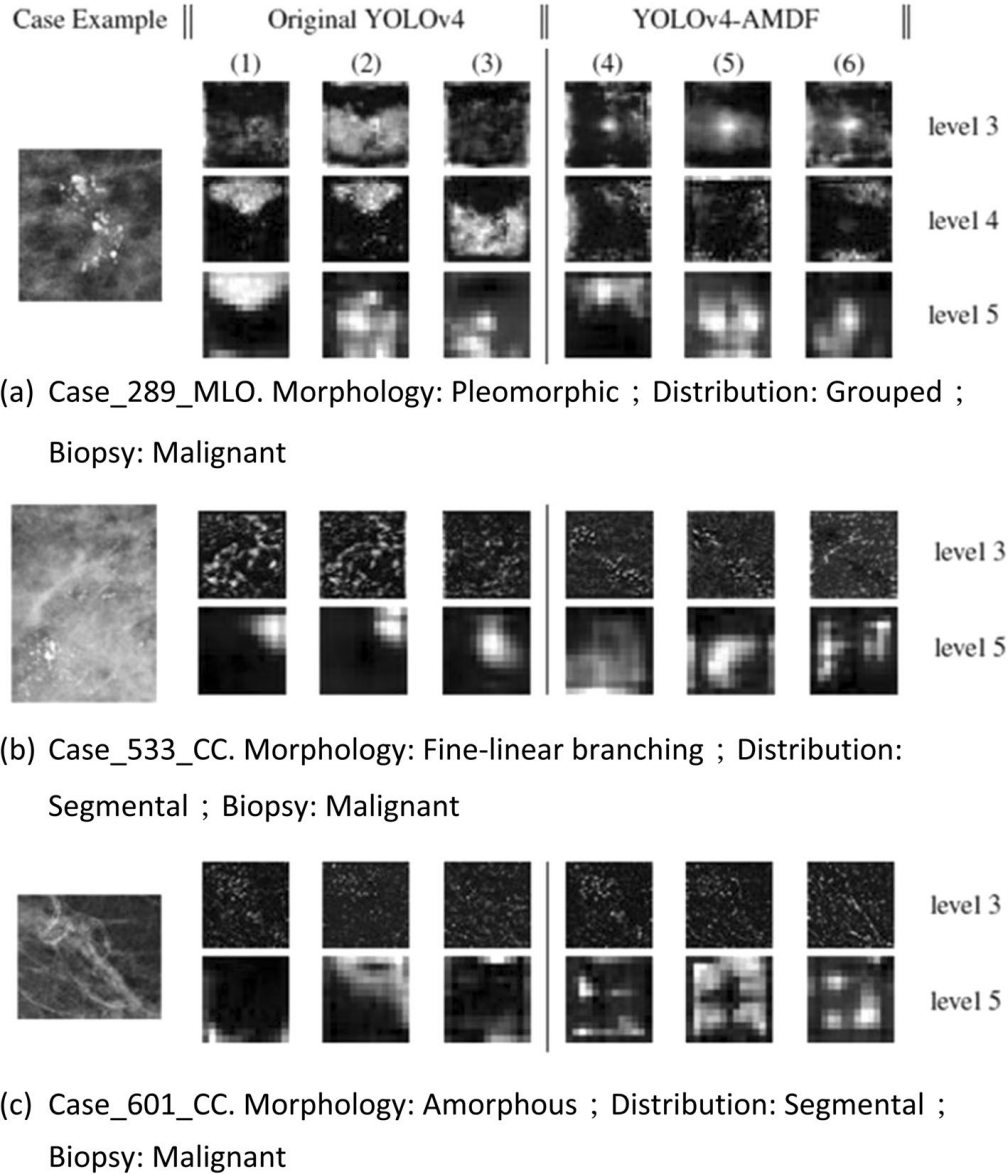
Visualization of YOLOv4 and YOLOv4-AMDF. **a** Case_289_MLO. Morphology: pleomorphic; distribution: grouped; biopsy: malignant. **b** Case_533_CC. Morphology: fine-linear branching; distribution: segmental; biopsy: malignant. **c** Case_601_CC. Morphology: amorphous; distribution: segmental; biopsy: malignant

We used visualization for the feature maps during the inference process of some cases from our in-house data set (Fig. 4). The original calcification regions are presented on the left column. The feature maps produced by YOLOv4 are displayed in the middle and the feature maps produced by YOLOv4-AMDF are displayed on the right column.

The loss function of YOLOv4-AMDF is binary cross-entropy and is represented as follows:

$$l\left(y,\widehat{y}\right)=-{\sum\limits_{i=1}^{n}}\left({y}_{i}log\left({\widehat{y}}_{i}\right)+\left(1-{y}_{i}\right)\mathrm{log}(1-{\widehat{y}}_{i})\right),$$

where *n* represents the number of samples; *ŷ* and *ŷi* represent the output of the model and the output of the model of sample *i*, respectively. *y* and *yi* is the ground truth and the ground truth of the sample *i*, respectively, and *yi* ∈ 0.1.

### Deep ensemble module for calcification classifier

The ensemble classifier was applied as the multi-layer perceptron (MLP). MLP with two hidden layers was trained using the adaptive learning rate optimization algorithm (Adam) solver, and hyperbolic tangent (Tanh) was used as the activation function. The initial learning rate was 0.005, and the regularization parameter alpha was 0.0001 (Table 5). The MLP input was composed of the confidence scores and the radiologists’ descriptors of the morphology and the distribution of calcifications. The confidence scores included output from two models, that is, preprocessed (mask overlap) images through YOLOv4, and original images through the proposed YOLOv4-AMDF, respectively. In our initial experiments, the clinical information including age, ratio and size of the region of interest were also included as part of the MLP. The mixed data type of input representation is adopted. The model confidence scores, age, size and aspect ratio of regions of interest containing calcifications are continuous variables, while the descriptors given by the radiologists are categorical variables. Additionally, we use one-hot encoding to represent the information of descriptions because the calcifications probably have more than one morphologic pattern. For the input with continuous variables, the values are scaled to have the data distribution with zero mean and unit variance before being input to MLP. The standard score of a sample x is calculated as:

$$z=\frac{x-u}{s},$$

where $u$ is the mean of the training samples and $s$ is the standard deviation of the training samples.

**Table 5:** MLP configuration

Hidden layer number	2
Activation	Tanh
Solver	Adam
Regularization parameter alpha	0.0001
Learning rate initialization	0.005
Validation fraction	0.2

### Performance evaluation of the proposed deep learning model

The initial model was pre-trained on the CBIS-DDSM dataset, and then retrained on the in-house dataset. The diagnostic performance of baseline YOLO, YOLO-AMDF, and the proposed AI system was estimated. We evaluated the performance of the model by using Monte Carlo cross-validation with a randomized split of in-house dataset into 85% for the train set and 15% for the test set for five holdout validations. When exploring AI performance on combined two views, that is, craniocaudal and mediolateral oblique views, or craniocaudal and mediolateral views, we considered the average scores of both images for each case.

To verify the effectiveness of our system, we tested it on the CBIS-DDSM and compared its performance with other algorithms applied to this dataset.

### Statistical analysis

The accuracy, sensitivity, specificity, positive predictive value (PPV), and receiver operating characteristic (ROC) analyses were performed, and area under the ROC curve (AUC) values for all the systems were obtained. The optimal cut-point value was determined using the method proposed by Ilker Unal [40]. *P* < 0.05 was set as statistically significant. Computation of *P* values and confidence intervals was conducted in Python using the Numpy (version 1.18.1) and Scipy (version 1.5.1) packages.

## Data Availability

The datasets generated and/or analyzed during the current study are not publicly available due to security of research data concerns but are available from the corresponding author on reasonable request.

## Abbreviations

AI: Artificial intelligence
BI-RADS: Breast Imaging Reporting and Data System
CBIS-DDSM: Curated Breast Imaging Subset of Digital Database for Screening Mammography
MLP: Multilayer perceptron
YOLO: You Only Look Once
